# ‘Location, Location, Location’: a spatial approach for rare variant analysis and an application to a study on non-syndromic cleft lip with or without cleft palate

**DOI:** 10.1093/bioinformatics/bts568

**Published:** 2012-10-08

**Authors:** Heide Fier, Sungho Won, Dmitry Prokopenko, Taofik AlChawa, Kerstin U. Ludwig, Rolf Fimmers, Edwin K. Silverman, Marcello Pagano, Elisabeth Mangold, Christoph Lange

**Affiliations:** ^1^Department of Genomic Mathematics, University of Bonn, 53127, Germany, ^2^Department of Applied Statistics, ^3^The Research Center for Data Science, Chung-Ang University, Seoul, 156-756, Korea, ^4^Institute of Human Genetics, ^5^Department of Genomics, Life and Brain Center, ^6^Institute for Medical Biometry, Informatics and Epidemiology, University of Bonn, Bonn 53127, Germany and ^7^Department of Biostatistics, Harvard School of Public Health, Boston, MA 02115, USA

## Abstract

**Motivation:** For the analysis of rare variants in sequence data, numerous approaches have been suggested. Fixed and flexible threshold approaches collapse the rare variant information of a genomic region into a test statistic with reduced dimensionality. Alternatively, the rare variant information can be combined in statistical frameworks that are based on suitable regression models, machine learning, etc. Although the existing approaches provide powerful tests that can incorporate information on allele frequencies and prior biological knowledge, differences in the spatial clustering of rare variants between cases and controls cannot be incorporated. Based on the assumption that deleterious variants and protective variants cluster or occur in different parts of the genomic region of interest, we propose a testing strategy for rare variants that builds on spatial cluster methodology and that guides the identification of the biological relevant segments of the region. Our approach does not require any assumption about the directions of the genetic effects.

**Results:** In simulation studies, we assess the power of the clustering approach and compare it with existing methodology. Our simulation results suggest that the clustering approach for rare variants is well powered, even in situations that are ideal for standard methods. The efficiency of our spatial clustering approach is not affected by the presence of rare variants that have opposite effect size directions. An application to a sequencing study for non-syndromic cleft lip with or without cleft palate (NSCL/P) demonstrates its practical relevance. The proposed testing strategy is applied to a genomic region on chromosome 15q13.3 that was implicated in NSCL/P etiology in a previous genome-wide association study, and its results are compared with standard approaches.

**Availability:** Source code and documentation for the implementation in R will be provided online. Currently, the R-implementation only supports genotype data. We currently are working on an extension for VCF files.

**Contact:** heide.fier@googlemail.com

## 1 INTRODUCTION

In the search for disease susceptibility loci (DSLs), genome-wide association studies (GWAS) have been a successful instrument for the identification of replicable genetic associations (Manolio *et al.,* 2008; Hardy and Singleton, 2009). They can interrogate almost the entire human genome for genetic associations. In GWAS, a large set of common variants, i.e. SNPs with high minor allele frequencies, is genotyped and tested for genetic association with the phenotype of interest. The SNPs that are genotyped on the panel of the GWAS SNP chips are typically selected so that they are strongly correlated with the SNPs that are not genotyped, enabling the indirect association testing of the untyped SNPs. However, for most complex diseases, the GWAS association signals are only able to explain a small fraction of the overall heritability that is predicted by classical heritability analysis (Visscher *et al.*, 2008). One possible explanation for this phenomenon is that many of the genetic associations that are detected by GWAS are caused by multiple rare DSLs, i.e. minor allele frequency of <1%, that are in proximity to one of the GWAS-SNPs (Goldstein, 2009; Manolio *et al.*, 2009). Because common variants are poor proxies for rare loci in association analysis or are not in linkage disequilibrium at all with rare disease-causing variants, it is difficult to identify and characterize rare DSLs in GWAS data. By recording all genetic loci of the region, high-throughput sequencing data contain the required information to address the rare variant hypothesis. Genomic regions that harbor disease-causing variants can be pinpointed and characterized.

Consequently, the arrival of high-throughput sequencing data for genetic studies of complex diseases poses a unique research opportunity for the localization of DSLs. At the same time, it constitutes a statistical challenge. Because the majority of the loci that are recorded by high-throughput sequencing are rare, classical single locus tests for genetic association, e.g. Amitrage-trend test (Lange and Laird, 2002), do not provide sufficient power for the underlying analysis questions. Collapsing methods have been suggested to address this problem. Using either a flexible or fixed thresholds for the minor allele frequencies of the loci that will be included in the analysis, such methods aggregate the genotypes of the rare variants in the genomic region for cases and controls, and construct a genetic association test between affection status and the genomic region. For example, one intuitive approach is the cohort allelic sums test (CAST) that counts the number of affected and unaffected individuals that are carriers of any rare variant in a given sample and predefined genetic region. Then, the fraction of carriers of a rare variant is compared with the fraction of non-carriers between cases and controls (Morgenthaler and Thilly, 2007). Li and Leal (2008) developed a Combined Multivariate and Collapsing (CMC) method that collapses rare variants into a single term and jointly assesses the effect of the collapsed rare variant term with the terms of common variants on a given trait using multivariate analysis. Other approaches have been suggested to account for potentially different effect sizes of the rare variants. Madsen and Browning (2009) proposed a weighted sum statistic in which loci are weighted according to their allele frequencies in the group of unaffected individuals. The weighted sum statistic approach has been subsequently extended by Price *et al.* (2010), who have generated the weights of the variants on the basis of external information. Ioanita-Laza *et al.* (2011) have proposed a method that compares the sharing patterns of rare alleles between cases and controls. Recently, general statistical frameworks have been developed (Ioanita-Laza *et al.*, 2011a; Neale *et al.*, 2011; Wu *et al.*, 2011) that, instead of collapsing the rare variant information, combine the information using suitable regression models, statistical learning methodology, etc. However, despite statistical challenges, the availability of rare variant/sequencing data offers the unique opportunity to identify DSLs.

So far, although highly biologically relevant, the existing methodology has ignored the information about the physical location of the rare variants. There are several reasons why physical proximity of rare variants in the genomic DNA sequence could be important. First, proteins can be composed of functional domains based on the amino acid sequence, and variants within the same protein functional domain, which may also be located in close proximity in the DNA sequence, could have similar impact on disease risk (Krebs *et al.*, 2011, Chapter 4, p. 90). Second, variants in the same gene regulatory element (e.g. enhancers, insulators, silencers and non-coding RNAs) would be physically clustered in the DNA sequence (Raab and Kamakaka, 2010). Finally, gene regulatory elements tend to cluster in certain genomic locations, such as the promoter region. More recently, Mathieson and McVean (2012) showed that the spatial distribution of rare variants can be used to depict population substructures. Based on the assumptions that deleterious rare variants and protective rare variants cluster together in different genomic regions, spatial clustering approaches can be used to construct powerful and robust association tests for rare variants. We develop such an approach that focuses on the physical position of the variants. The approach thereby does not require prior knowledge about biologically relevant segments in the genomic region of interest and about the effect size directions of the different alleles. The methodology is computationally fast, allowing applications to whole genome sequencing studies. We assess the power of our approach based on simulation studies and compare it with existing methodology. In the presence of DSL clusters, the proposed approaches achieve substantially higher power levels than standard methods. This is especially true for scenarios in which deleterious and protective variants are present. Nevertheless, in the absence of DSL clusters, they perform as well as standard methods. The capabilities of the approach are illustrated by an application to a sequencing study of the genomic region that was identified by a GWAS for non-syndromic cleft lip with or without palate. Our derived distance measure yields a highly significant association between affection status and the genomic region, whereas standard collapsing and weighting approaches do not provide significant results.

## 2 METHODS

For the analysis of rare variants, a biologically plausible hypothesis is that alleles of rare variants that have the same type of effect on disease risk, e.g. either deleterious or protective, occur in the same part of the genomic region of interest. Because the parts of the genomic region that are relevant for changes in disease risk are typically not known before the analysis, our goal is to construct an association test for rare variant analysis that identifies clusters of rare alleles and examines their effects on disease risk. In the analysis of spatial data (Kowalski *et al.*, 2002; Bonetti and Pagano, 2005), the distribution of the physical distances between events is used to detect the spatial clustering of events. We will apply the same idea here to the analysis of rare variants and their genotypes.

In a first step, we identify the cumulative frequencies of all detected variants in cases and controls, and apply inverse frequency weights to each detected rare variant. We then combine the information on the locations of the rare variants in the cases and in the controls, and derive the distribution functions of genomic distances between the rare alleles in both groups. Next, we construct a test statistic that is suitable to capture differences between the distance distribution functions for the two groups. Because the test statistic will be driven by the different clustering of the variant locations in the case and control groups, the power of the test statistic is not negatively influenced by the presence of different effect directions in the region, i.e. deleterious alleles and protective alleles.

Because the hypothesis is that DSLs with the same effect direction cluster in the same genomic region, the differences between the distribution functions are particularly of interest for the small genomic distances. We therefore introduce a test that captures the information of both, the degree of skewness of the two allelic distributions towards small distances and the systematic differences of the actual physical positions of the rare variants between cases and controls. The statistical significance of the discussed test statistic is obtained by permutations that randomly assign case/control status to the study population while maintaining the total number of cases and controls.

We assume that a defined genomic region has been sequenced in 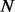 subjects in the context of a case–control study, recording the physical position on a total of 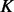 rare variants and their genotypes. We denote each detected rare variant as 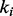 with 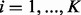.

Every variant 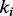 shows a minor allele frequency that is smaller than a pre-specified threshold value. Because, for rare variant analysis, there is usually not sufficient power to detect genetic associations with a single locus, we combine the information about the frequencies and locations of the rare variants over the disease status in our sample.

Each detected variant 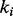 is also associated with a physical position 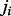, and 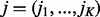 represents the vector that contains all physical positions of the 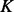 variants in the sample in ascending order.

The allele frequency of each rare variant can be estimated separately in cases and controls. We define 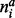 to be the number of rare alleles in cases at variant 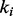, and 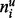 to be the respective observed number of rare alleles in controls.

We receive a total of 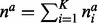 rare alleles in the cases and a total of 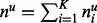 rare alleles in the controls.

To emphasize the importance of spatial proximity between variants and to control for an uneven distribution of the allelic frequencies, we define weights that are both cluster and frequency dependent. The physical positions of the variants are incorporated into the weights by determining the distance to the nearest neighbor variant for each variant 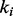 in the sorted positional vector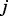 that contains the physical positions of all detected variants:

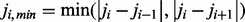



Madsen and Browning (2009) and Ionita-Laza *et al.* (2011b) have shown that depending on whether a variant has a disease-causing or protective effect, efficient weighting schemes can be constructed based on the allele frequency of the rare variants in only either controls or cases. For cases and controls, we define separately inverse frequency weights that weigh variants based on their minor allele frequency and combine these frequency weights with the information about the physical position of the variants. Our weighting scheme for each detected variant 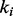 based on the distribution of variants in cases, is given by:

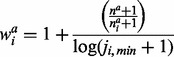



The weighting scheme for each variant 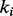 based on the distribution of the variants in controls is defined as:

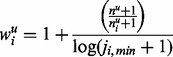



Thus, for a variant 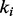, the rounded to integer allele counts in cases and controls weighted by the distribution of variants in cases are given by:

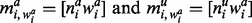



And accordingly for a given variant 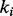, the rounded to integer allele counts for cases and controls weighted by the distribution of variants in controls are given by:

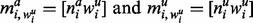



In the next step, we create sequences where the physical positions 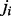 of variants 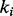 are replicated according to the detected weighted allele counts.

The variant position sequences for cases and controls weighted according to the distribution of variants in cases are given by:

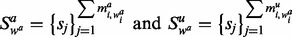



And synonymously we derive the position sequences for cases and controls weighted on the distribution of variants in controls as:

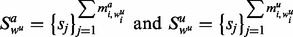



Our goal is to construct a test statistic that assesses the spatial proximity between variants of each group. For variants that increase disease risk, we assume that their locations tend to be spatially clustered in a certain part of the genomic region. Variants that are protective or have no effect on disease risk are assumed to either cluster in a different part of the genomic region or have a weaker or no tendency to cluster.

For each weighting scheme, we derive the distributions of the rare allele distances in cases and controls. We obtain the distances between two adjacent alleles by subtracting the variant location of one allele from the variant location with the next larger position order. It is important to note that, if a rare allele at one locus is observed multiple times in cases or controls, then the corresponding distances between them are zero.

For the weighting scheme based on the frequencies of variants in cases, we receive the following distance vectors for cases and controls:

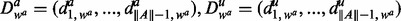

with the elements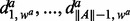 and 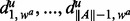 representing the derived distances between the weighted variant positions.

And accordingly, for the weighting scheme that incorporates the variant allele frequencies of controls, we can display the distance vectors of cases and controls as:

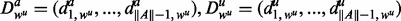



It is important to note that the applied weighting schemes thereby solely influence the skewness of the derived distance distribution functions, but have no impact on the values of the observed non-zero distances.

Based on the allelic distance distributions in cases and controls, we now construct our location-based association test for rare variant data, and apply it separately for each weighting scheme. In the test statistic, we want to incorporate the information about both, the allele frequencies of the rare variants and the physical distances between the rare variants; the statistic has to take into account both the skewness of the distance distribution functions and the variance of the derived distances for cases and controls. In most settings, the derived distance distribution functions will be highly right skewed and have the same median so that rank-based tests that rely on the median (e.g. Wilcoxon rank sum test) will not provide efficient power. Similarly, non-parametric tests that directly rely on the shape of the distance distribution functions (e.g. Kolmogorov–Smirnov test) will concentrate on the difference in the skewness of the distance distribution functions. However, they ignore information about the actual physical distances between the rare variants.

One alternative approach to directly compare the two weighted distance distribution functions is the application of a non-parametric two-sample test on the variability of the distance distribution functions. Ansari and Bradley (1960) have developed such a test that directly examines the dispersion of two independent distribution functions—
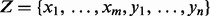
—and sorts the values in an increasing order so that 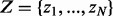. The function 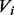 displays the distribution of ranked positions from both samples with 
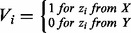


The Ansari–Bradley test statistic can then be expressed as

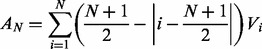



The Ansari–Bradley test assumes the same location parameter for the tested two independent distribution functions and tests for differences in the ratio of scales. The scale of a distribution function describes its spread, i.e. the scale of a normal distribution is defined by its variance.

Because we apply two different weighting schemes on each sample, we obtain two test statistics for the Ansari–Bradley test: one test statistic for the distance distribution functions weighted on the distribution of variants in cases and another one for the distance distribution functions weighted on the distribution of variants in controls.

We define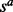 to be the ratio of scales weighted by the distribution of variants in cases and 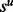 to be the ratio of scales of the two derived distance distribution functions weighted on the distribution of variants in controls.

For both weighting schemes, we independently test whether the ratio of scales (*s*) of the weighted distance distribution functions for cases and controls is equal to one.

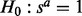
 versus 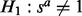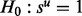 versus 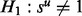

We select the maximum of both test statistics based on our hypotheses (1) and (2) as our final test statistic.

A rejection of the null hypothesis implies an association of the tested rare variants in the region with affection status. Because of the likely presence of tied observations in the distance distribution functions, we use an implementation of the standard Streitberg/Roehmel shift algorithm to retrieve exact distribution functions for our test statistics (Streitberg and Roehmel, 1986).

The significance of the test statistic is obtained based on permutations. In each replicate, case–control status is randomly assigned to each proband in such a way that the total number of cases and controls of the original study is maintained. The genotypes of the probands are kept fixed, maintaining the LD structure of the region in the permutation sample. The *P*-value is estimated as the proportion of permutation test statistics, which are more “extreme” than the actually observed test statistic for the real data.

For the application of our approach to dosage data, i.e. those datasets that contain genotype probabilities instead of allele counts, we recommend calculating the expected allele count for each subject and variant 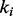 on the basis of the genotype probabilities and applying our method without modifications.

Using simulation studies, we evaluate the performance and power of the Ansari–Bradley test based on the weighted distance distribution functions. We also apply our analysis approach to a sequencing dataset for non-syndromic cleft lip with or without palate to demonstrate its practical relevance.

## 3 RESULTS

### 3.1 Simulation study

#### 3.1.1 Generation of the data

For the generation of the genetic data in the simulation study, we implemented the model as described in the simulation study by Ionita-Laza *et al.* (2011b). We defined the mutation rate to be 1.5 × 10^−8^ and simulated 5000 haplotypes with 500 000 base pairs. Subsequently, we selected 30 SNPs with a predefined upper MAF cutoff. Based on two randomly chosen haplotypes, we used an additive disease model with a disease prevalence of 0.15 to derive our genotypes. We assigned an invariant risk ratio of 3.0 to each SNP that has a causal influence on the affection status. We repeated the last step for each specification until we received a sample of 500/500 cases and controls or 750/750 cases and controls.

#### 3.1.2 Specifications of simulation runs

We applied different simulation scenarios to examine the type 1 error and power of our proposed methods.

In the following, we refer to the proposed rare variant test statistics based on the allelic distances as distance-based measure (DBM).

Moreover, we compare the proposed test statistic with some of the existing statistical methods that relate rare variants to a dichotomous phenotype: the CMC approach (Li and Leal, 2008) that assigns equal weights to each rare variant, two methods that use weighting schemes based on allele frequencies or prior biological knowledge (Madsen and Browning, 2009; Price *et al.*, 2010), the replication-based strategy of Ionita-Laza *et al.* (2011b) that suggests a weighting scheme based on those alleles that are present more often in cases compared with controls and two flexible regression-based methods that test the variance of effects for a given set of rare variants (Neale *et al.*, 2011; Wu *et al.*, 2011).

We denote the test statistic of Li and Leal (2008) as CMC, the test statistic of Madsen and Browning (2009) as MB, the test statistic of Price *et al.* (2010) as Price, the test statistic of Ionita-Laza *et al.* (2011b) as RB, the test statistic of Wu *et al.* (2011) as SKAT and the test statistic of Neale *et al.* (2011) as C-Alpha.

For the simulations, we first differentiated between two different settings that relate to the physical distribution of the rare variants in the derived samples. In the clustered scenario (Clustered = Yes), all variants that have a disease-causing effect are sampled as a sequence with close physical close position to each other, whereas the remaining non-causative/protective variants and their physical positions are randomly sampled outside this sequence. In the non-clustered scenario (Clustered = No), the physical position of all selected variants is randomly assigned. The degree of clustering of the simulated variants, i.e. the distances between the associated loci, was thereby chosen according to the observed degree of clustering for the real dataset that we used for the application of our method (Section 3.2).

Next, we varied the sample size between 500/500 cases and controls or 750/750 cases and controls. In all derived different scenarios, we examined two different upper MAF thresholds, namely 1% and 0.5%, to evaluate the sensitivity of the presented measures to different MAF cutoffs. We used 1000 replicates to evaluate the type 1 error and 500 replicates to estimate the power of each approach. All showed estimates result from two-sided testing. The *P*-values used for the type 1 error evaluation and for the power estimates are based on 1000 permutations.

#### 3.1.3 Evaluation of type 1 error

Table 1 shows the simulation results for the type 1 error for the outlined scenarios. We chose two different significance levels—*α* = 0.05 and *α* = 0.01—to evaluate the type 1 error. The simulation study results suggest that our approach maintains the type 1 error.

**Table 1.** Evaluation of type 1 error (500/500 cases/controls and 750/750 cases/controls, 30 rare variants)

MAF	*α*	Number of cases/controls	DBM
0.01	0.05	500/500	0.051
0.01	0.01	500/500	0.008
0.005	0.05	500/500	0.050
0.005	0.01	500/500	0.015
0.01	0.05	750/750	0.047
0.01	0.01	750/750	0.008
0.005	0.05	750/750	0.050
0.005	0.01	750/750	0.011

Tested at *α* = 0.05 or *α* = 0.01, 1000 replicates.

#### 3.1.4 Power estimates

For the power estimates of the methods, we included one additional specification in the outlined scenarios. In a first setting, we assumed that, for all 10 DSLs, the rare allele increases the disease risk. In a second setting, we assumed the simultaneous presence of deleterious and protective effects, i.e the rare alleles of seven DSLs are disease causing, whereas the rare alleles of three DSLs have a protective effect.

Based on 500 replicates, Table 2 shows the power estimates between the affection status and the sequenced rare variants in a non-clustered scenario for the following methods: CMC, Price, MB, RB, SKAT, C-Alpha and DBM.

**Table 2.** Power estimates of outlined approaches in a non-clustered scenario

MAF	0.01	0.005	0.01	0.005	0.01	0.005	0.01	0.005
Number of cases/controls	500/500	500/500	750/750	750/750	500/500	500/500	750/750	750/750
Number of variants	30	30	30	30	30	30	30	30
Number of risk variants/number of protective variants	10/0	10/0	10/0	10/0	7/3	7/3	7/3	7/3
CMC	0.222	0.148	0.280	0.176	0.094	0.054	0.108	0.070
Price	0.232	0.152	0.274	0.160	0.132	0.102	0.160	0.140
MB	0.248	0.176	0.330	0.194	0.104	0.066	0.128	0.100
RB	0.372	0.250	0.446	0.288	0.166	0.114	0.226	0.168
SKAT	0.254	0.176	0.346	0.176	0.128	0.074	0.162	0.114
C-Alpha	0.172	0.112	0.244	0.134	0.130	0.104	0.210	0.126
DBM	0.332	0.218	0.342	0.238	0.132	0.122	0.152	0.162

Tested at *α* = 0.05, 500 replicates.

The power estimates for the non-clustered scenario show that our constructed DBM had a better performance than the collapsing approach (CMC), the two methods based on weighting schemes (Price, MB) and the SKAT and C-Alpha test, when the effect direction of the causative rare variants is the same.

In the case of mixed-effect directions of the causative variants, the SKAT and C-Alpha test showed more power in one of the four outlined scenarios compared with the DBM method.

The replication-based measure (RB), however, outperformed all other compared methods (CMC, Price, MB,SKAT and C-Alpha) in all specified simulation runs in the non-clustered scenario. For a rather small MAF cutoff (MAF < 0.005), the power advantage of the RB method compared with our DBM for the non-clustered scenario became similar though. In general, the simulation studies suggest that our approach achieves power levels that are comparable with the power levels of current methodology, even in scenarios where there is no clustering of variants.

Table 3 provides the power estimates of the outlined specifications (10 DSLs that increase the disease risk, and accordingly 7 risk DSLs and 3 protective DSLs) and specified methods (CMC, Price, MB, RB, SKAT, C-Alpha and DBM) for a clustered scenario. For the clustered scenario, the power estimates are also based on 500 replicates.

**Table 3.** Power estimates of outlined approaches in a clustered scenario

MAF	0.01	0.005	0.01	0.005	0.01	0.005	0.01	0.005
Numberof cases/controls	500/500	500/500	750/750	750/750	500/500	500/500	750/750	750/750
Number of variants	30	30	30	30	30	30	30	30
Number of risk variants/number of protective variants	10/0	10/0	10/0	10/0	7/3	7/3	7/3	7/3
CMC	0.210	0.122	0.308	0.170	0.094	0.082	0.128	0.070
Price	0.242	0.166	0.290	0.196	0.162	0.090	0.162	0.128
MB	0.234	0.142	0.342	0.182	0.112	0.096	0.148	0.084
RB	0.326	0.214	0.488	0.292	0.186	0.142	0.236	0.170
SKAT	0.230	0.148	0.332	0.194	0.130	0.102	0.162	0.112
C-Alpha	0.190	0.096	0.218	0.136	0.156	0.084	0.206	0.114
DBM	0.392	0.294	0.494	0.352	0.310	0.208	0.324	0.224

Tested at *α* = 0.05, 500 replicates.

Because all compared methods except the DBM method are not sensitive to location clustering of the variants, the power estimates of CMC, Price, MB, RB, SKAT and C-Alpha did not differ considerably in the clustered and non-clustered scenario.

Our constructed distance-based measure (DBM), however, is sensitive to physical closeness of variants, and thus showed far more power in a clustered scenario than in a non-clustered scenario. This power gain had the consequence that in the clustered scenario our distance-based measure (DBM) showed the highest power estimates in all simulation runs compared with the other methods (CMC, Price, MB, RB, SKAT and C-Alpha).

The power advantages of the DBM method compared with the replication-based measure (RB) ranged from 1 to ∼35% when only risk variants are present in the simulated genomic region. The power advantage of the DBM measure was thereby greater for the smaller MAF-cut off. When both risk and protective variants are defined in the simulation runs, our newly introduced measure showed even more power compared with the other methods. In one simulated setting, the DBM measure had a power advantage of >60% compared with the next best performing method (RB). It is important to note, however, that the power estimates of all outlined methods were reduced in simulation runs where variants with opposed effect directions were included.

In addition, we also re-ran some of our simulations with a constant genetic attributable risk to confirm our qualitative conclusion about the performance of the different methods (data not shown).

### 3.2 Application of the discussed approach to a sequencing dataset on non-syndromic cleft lip with or without cleft palate

We applied the discussed approach to a sequencing study on non-syndromic cleft lip with or without cleft palate (NSCL/P). The dataset was generated by a follow-up sequencing of 96 cases and 96 controls in gremlin-1 (GREM1), a candidate gene located in a genomic region at 15q13.3 that was identified as a suggestive NSCL/P locus in a GWAS by Mangold *et al.* (2010). GREM1 is coding for a known antagonist of the bone morphogenic protein 4 (BMP4). BMP4 has been shown to regulate mammalian palatogenesis (Zhang *et al.*, 2002) and has been reported to be associated with orofacial clefting in humans (Suzuki *et al.*, 2009). The follow-up sequencing of both 5' and 3' UTR, as well as coding regions GREM1, resulted in a discovery of 27 variants with an MAF between 0.003 and 0.573.

**Fig. 1.**
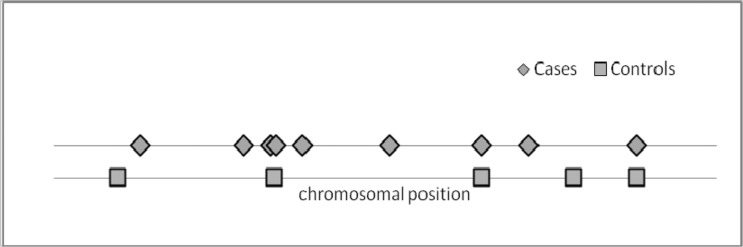
Spatial distribution of rare variants in the sample. Two rare variants (one rare variant in cases and another in controls) with outlying positions are not shown in the figure

Figure 1 depicts the spatial distribution of the 14 detected rare variants (MAF ≤ 0.05), with an MAF range of 0.003–0.029 for cases and controls. Although the discovered rare variants in controls are rather equally distributed on the chromosome, it can be seen for the groups of cases that four rare variants tend to cluster.

For our proposed distance-based approach (DBM), we select two different MAF cutoffs (1 and 5%) and compared them with the other methods (CMC, RB, Price and MB). Table 4 shows the *P-*values of our derived distance-based test statistic (DBM) compared with the other outlined collapsing and weighting methods. For all compared methods, the *P*-values are based on 1000 permutations.

**Table 4.** *P*-values of the compared methods for testing the association of 15q13.3 with NSCL/P

MAF	CMC	Price	MB	RB	SKAT	C-Alpha	DBM
0.01	0.783	0.279	0.562	0.256	0.062	0.071	0.006
0.05	0.695	0.281	0.556	0.331	0.152	0.914	0.011

*P*-values are based on 1000 permutations.

It can be seen that our newly introduced measure (DBM) finds a highly significant association of the sequenced 15q13.3 region at both defined upper MAF cutoffs, whereas the other methods (CMC, Price, MB, RB, SKAT and C-Alpha) fail to detect a significant association at a 5% significance level. Before the application of our method to real data, one should perform simulation studies to determine a suitable window size as for the other mentioned rare variant association tests, given the sample size and the assumption of the disease parameters, i.e. prevalence, effect size, etc. For the application of our variant position-based test, the window size should be chosen according to the spatial distribution of the variants on the chromosome, so that variant clusters are not separated.

## 4 CONCLUSION

So far, existing statistical methodology for association analysis has ignored the physical locations of the variants. In this communication, we have proposed a class of methods to test the association of rare variants to a dichotomous trait that incorporates the underlying spatial distribution structure of the rare variants. Our method is based on statistical clustering methodology. Instead of collapsing or combing the genotypes of rare variants in the genomic region of interest, our test statistic takes advantages of physical distances/locations of the alleles at the rare variant loci and detects rare variant patterns that are different between cases and controls. As a result, we obtain a new class of association tests for rare variant analysis that can aid the localization of the biological relevant segments in the analyzed genomic region. As for any rare variant approach, subpopulation-specific patterns in the variant distribution can bias the analysis results of our test statistic. Although the findings of Mathieson and McVean (2012) suggest that reasonable amounts of subpopulation-specific variant distributions do not severely affect the existing rare variant analysis approaches, careful QC of the data, i.e. detection of population substructure and outlier-removal accordingly, is mandatory before the application of our approach. One approach here could be to apply our testing strategy to known null regions, i.e. regions without any genetic effects, and compare the performance of the test statistic in these regions with the genomic region of interest. The detection of population substructure is especially important, if samples from other studies or sources, such as the 1000 Genome Project, are included in the analysis to increase the statistical power.

It is important to note, however, that our method is best suited for high coverage sequencing data to detect and test the spatial structure of variants. SNP data that were obtained from GWAS SNP chips, because of the pre-defined SNP locations on such chips, offer only limited information on the spatial distribution of variants in a genomic region. Moreover, the presented method also has limitations in testing aggregated, but positional unconnected, genome regions, like non-coding regions.

For now, we focus on a very intuitive test statistic that compares the two distance distributions between cases and controls, using a Ansari–Bradley test statistic. Currently, we are working on an extension of the approach that allows the integration of covariates in the test statistic and to generalizations to quantitative traits.

*Funding*: The project described was supported by Award Number (R01MH081862, R01MH087590) from the National Institute of Mental Health and Award Number (U01HL089856, U01HL089897) from the National Heart, Lung, and Blood Institute.

*Conflict of Interest*: none declared.

## References

[bts568-B1] Ansari A, Bradley R (1960). Rank-sum tests for dispersions. Ann. Math. Statist.

[bts568-B2] Bonetti M, Pagano M (2005). The interpoint distance distribution as a descriptor of point patterns, with an application to spatial disease clustering. Stat. Med.

[bts568-B3] Goldstein DB (2009). Common genetic variation and human traits. N. Engl. J. Med.

[bts568-B4] Hardy J, Singleton A (2009). Genomewide association studies and human disease. N. Engl. J. Med.

[bts568-B5] Ionita-Laza I (2011a). Finding disease variants in mendelian disorders by using sequence data: methods and applications. Am. J. Hum. Genet.

[bts568-B6] Ionita-Laza I (2011b). A new testing strategy to identify rare variants with either risk or protective effect on disease. PLoS Genet..

[bts568-B7] Kowalski J (2002). A nonparametric test of gene region heterogeneity associated with phenotype. J. Am. Stat. Assoc..

[bts568-B8] Krebs JE (2011). Lewin’s GENES X.

[bts568-B9] Lange C, Laird NM (2002). Power calculations for a general class of family-based association tests: dichotomous traits. Am. J. Hum. Genet.

[bts568-B10] Li B, Leal SM (2008). Methods for detecting associations with rare variants for common diseases: application to analysis of sequence data. Am. J. Hum. Genet.

[bts568-B11] Madsen BE, Browning SR (2009). A groupwise association test for rare mutations using a weighted sum statistic. PLoS Genet..

[bts568-B12] Mangold E (2010). Genome-wide association study identifies two susceptibility loci for nonsyndromic cleft lip with or without palate. Nat. Genet.

[bts568-B13] Manolio TA (2008). A HapMap harvest of insights into the genetics of common disease. J. Clin. Invest.

[bts568-B14] Manolio TA (2009). Finding the missing heritability of complex diseases. Nature.

[bts568-B15] Mathieson I, McVean G (2012). Differential confounding of rare and common variants in spatially structured populations. Nat. Genet.

[bts568-B16] Morgenthaler S, Thilly WG (2007). A strategy to discover genes that carry multiallelic or mono-allelic risk for common diseases: a cohort allelic sums test (CAST). Mutat. Res.

[bts568-B17] Neale BM (2011). Testing for an unusual distribution of rare variants. PLoS Genet.

[bts568-B18] Price AL (2010). Pooled association tests for rare variants in exonresequencing studies. Am. J. Hum. Genet.

[bts568-B19] Raab JR, Kamakaka RT (2010). Insulators and promoters: closer than we think. Nat. Rev. Genet.

[bts568-B20] Streitberg B, Roehmel J (1986). Exact distribution for permutation and rank tests: an introduction to some recently published algorithms. Statist. Software Newsletter.

[bts568-B21] Suzuki S (2009). Mutations in BMP4 are associated with subepithelial, microform, and overt cleft lip. Am. J. Hum. Genet.

[bts568-B22] Visscher PM (2008). Heritability in the genomics era–concepts and misconceptions. Nat. Rev. Genet.

[bts568-B23] Wu MC (2011). Rare-variant association testing for sequencing data with the sequence Kernel association test. Am. J. Hum. Genet.

[bts568-B24] Zhang Z (2002). Rescue of cleft palate in Msx1-deficient mice by transgenic Bmp4 reveals a network of BMP and Shh signaling in the regulation of mammalian palatogenesis. Development.

